# Uncoupling of T Cell Receptor Zeta Chain Function during the Induction of Anergy by the Superantigen, Staphylococcal Enterotoxin A

**DOI:** 10.3390/toxins2071704

**Published:** 2010-06-30

**Authors:** William D. Cornwell, Thomas J. Rogers

**Affiliations:** FELS Institute, Temple University School of Medicine, 3307 North Broad Street, Philadelphia, PA 19140, USA; Email: cornwell@temple.edu (W.D.C.)

**Keywords:** superantigen, *Staphylococcus aureus*, SEA, anergy, zeta chain, enterotoxin

## Abstract

*Staphylococcus aureus* enterotoxins have immunomodulatory properties. In this study, we show that Staphylococcal enterotoxin A (SEA) induces a strong proliferative response in a murine T cell clone independent of MHC class II bearing cells. SEA stimulation also induces a state of hypo-responsiveness (anergy). We characterized the components of the T cell receptor (TCR) during induction of anergy by SEA. Most interestingly, TCR zeta chain phosphorylation was absent under SEA anergizing conditions, which suggests an uncoupling of zeta chain function. We characterize here a model system for studying anergy in the absence of confounding costimulatory signals.

## 1. Introduction

*Staphylococcus aureus* enterotoxins have been implicated in a number of acute and chronic human diseases including food poisoning, tampon related toxic shock, scalded skin syndrome, Kawasaki syndrome, and shock [1,2,3]. The toxins produced by *S. aureus* include Staphylococcal enterotoxin A (SEA), B, C_1_, C_2_, C_3_, D, E, G-R, U, and toxic shock syndrome toxin-1 (TSST-1). Collectively, these toxins are classified as superantigens since they bind to major histocompatibility complex (MHC) class II molecules and are recognized by essentially all T cells expressing certain T cell receptor (TCR) variable β (Vβ) alleles [1,2]. This suggests that up to 20% of the T cell repertoire could be activated by a single superantigen. Finally, another characteristic which distinguishes a superantigen from a normal antigen is the lack of a requirement for processing by the antigen presenting cell (APC). 

Complete T cell activation requires the recognition of antigen presented in the context of MHC class I or II to the TCR and the delivery of costimulatory signals via either the interaction of CD28 with B7-1/-2, or by the activation of cytokine receptors. The first of these signals is mediated via the TCR which is a complex of proteins consisting of the α and β heterodimer along with the γ, δ, and ε chains of CD3 and a homodimer of the ζ chain. These subunits of CD3 as well as the ζ chain have been shown to possess cytoplasmic tails which become rapidly phosphorylated upon normal stimulation of the TCR [4]. In addition, kinases, such as, p56lck, p59fyn, and zap-70 are brought in close proximity of the TCR by CD4 and other costimulatory molecules and are rapidly phosphorylated following TCR stimulation [5,6,7,8,9,10,11]. The results of these signaling events are the production of IL-2, the autocrine IL-2-induced activation of the IL-2 receptor complex, and clonal expansion of the T cell through proliferation.

However, an incomplete or partial stimulation of T cells via the TCR in the absence of costimulatory signals results in a state of tolerance or anergy [12]. Anergy can be induced by several different methods. The first described induction of anergy involved complete TCR stimulation in the presence of neutralizing antibodies to IL-2 and IL-2 receptor [13]. It was suggested that conditions which favor anergy induction are insufficient for normal IL-2 production and the absence of autocrine stimulation by IL-2 results in the development of anergy. Furthermore, Boussiotis *et al.* [14] demonstrated that stimulation of human T cells with antigen in the absence of CD28 signaling, but in the presence of IL-2, IL-4, or IL-7, inhibited the development of anergy. Other models employed cross-linking of CD3 in the absence of costimulation, or peptide with a single amino acid substitution (altered peptide ligand; APL) presented by live APCs to induce anergy [11,15,16,17]. Our laboratory and others have demonstrated that superantigens can induce anergy in the absence of MHC class II bearing cells in murine and human T cells [18,19,20]. Interestingly, superantigen-induced anergy was not avoided when exogenous IL-2 was present [19,20]. This is a fundamental difference between *in vitro* models of anergy induction where antigen is presented by functionally impaired APCs. Similarly, several investigators have demonstrated anergy induction following superantigen administration *in vivo* [21,22,23,24,25]. These data suggest that, in contrast to normal antigenic stimulation, superantigens may provide a distinct and physiologically relevant signal(s) via the TCR to induce a state of anergy. 

Here we report an *in vitro* model of anergy using superantigen in the absence of MHC class II bearing cells to examine the proximal TCR signaling events that lead to the induction of an anergic phenotype. The advantage of this experimental system is that T cell clonal anergy is induced under highly simplified conditions where there are no complicating signals contributed by the APCs. The purpose of the present studies was to characterize the proximal signaling events following induction of anergy under these conditions. In this system, the A.E7 clone was anergized by SEA in the absence of APCs, while the cells undergo a dose-dependent proliferative response, and remain responsive to IL-2. We find that p56lck expression, but not p59fyn or ZAP-70, is reduced rapidly following anergy induction with SEA alone. Importantly, under these conditions, the zeta chain of the TCR failed to become hyper-phosphorylated, in contrast to conditions of normal T cell activation. Finally, these studies demonstrate that the failure to hyper-phosphorylate zeta chain is mediated directly by the ligation of the TCR by superantigen. 

## 2. Results and Discussion

### 2.1. The role of APCs in the SEA-induced proliferation of the A.E7 T cell clone

**Figure 1:**
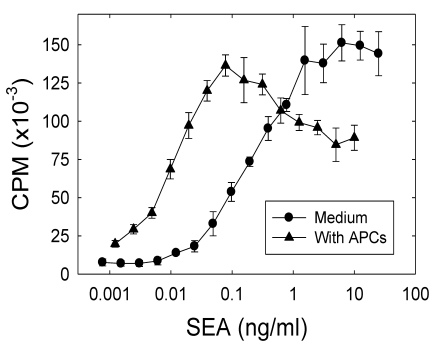
A.E7 proliferative response to SEA. A.E7 cells were cultured with the designated concentrations of SEA for 48 hours in the presence (▲) or absence (●) of irradiated B10.A splenocytes (2.5 × 10^5^ cells/culture). The proliferative response was assessed after 48 hours. The results are representative of more than three independent experiments. The error bars represent standard error. The background proliferation of the A.E7 clone was 168 CPM in this experiment.

We wanted to determine the capacity of A.E7 T cells to proliferate in response to SEA in the presence or absence of syngeneic class II-bearing antigen presenting cells (APC). The A.E7 cells were stimulated for two days in culture with SEA (0.001–25 ng/mL) with or without irradiated B10.A splenocytes. The results in Figure 1 demonstrate a strong proliferative response of the A.E7 T cell clone stimulated with SEA (EC50 approximately 0.01 ng/mL) presented by APC. Furthermore, in the absence of APCs, SEA also induces a comparable level of proliferation in the A.E7 T cell clone (EC50 approximately 0.2 ng/mL), however the response in the absence of APCs requires approximately 20-fold higher concentration of SEA. These results are consistent with previous results reported from this laboratory [18,19], and others [26,27], which have shown that T cells may exhibit a proliferative response to superantigen in the absence of intact APCs.

**Figure 2:**
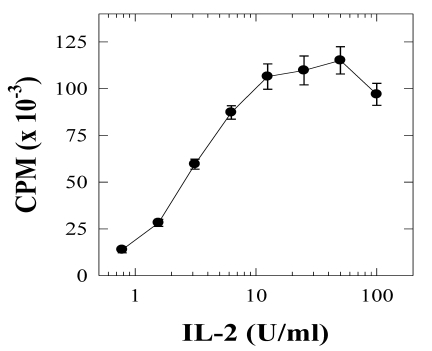
Proliferative response of the A.E7 clone to IL-2 stimulation. A.E7 cells were cultured with the designated concentrations of IL-2 for 48 hours, and the proliferation was determined by [3H]-thymidine incorporation. Background proliferation was 283 CPM for this experiment. These results are representative of two experiments. The error bars represent standard error.

In addition, the A.E7 T cell clone can respond to other stimuli, including IL-2. We tested the response of the A.E7 cells to IL-2 in the absence of APCs, and the results show a strong dose-dependent proliferative response (Figure 2). The cells were cultured with IL-2 (0.7–100 U/mL) for 48 hours in the absence of any other stimulus. IL-2 induced a proliferative response in the A.E7 cells comparable to normal antigenic stimulation. These results demonstrate that resting A.E7 cells have an intact IL-2 receptor and IL-2 receptor signaling pathway(s). Furthermore, these data are consistent with previously published observations that T cell proliferation alone cannot prevent anergy induction [28]. 

### 2.2. SEA-induced T cell anergy

**Figure 3:**
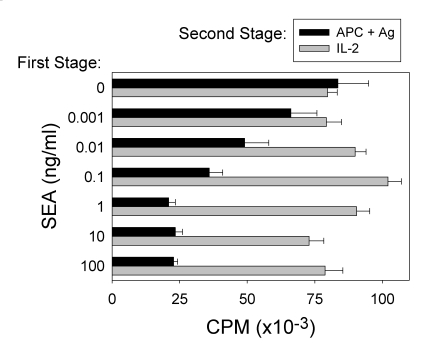
Dose-dependent SEA-induced anergy induction in the A.E7 clone. A.E7 cells were stimulated for 48 hours in culture (first stage) with SEA in the absence of APCs. The cells were washed and re-cultured for 48 hours in medium alone. The A.E7 cells were cultured for 48 hours (second stage) with either irradiated APCs (black bars) presenting peptide antigen (10 μM) or IL-2 (20 U/mL; gray bars). These data are representative of more than three independent experiments. The error bars represent standard error. The background proliferation of the A.E7 cells was 1,290 CPM.

In an effort to examine the functional activity of the stimulated A.E7 cells, the cells were stimulated with SEA in the absence of MHC class II-bearing cells (Figure 3). After two days in this first stage of culture with SEA, the cells were washed and re-cultured for two to four days in fresh medium to allow the clone to rest. In the second stage of the experiment, the ability of the cells to proliferate in response either to peptide antigen or SEA presented by irradiated APCs or to exogenous IL-2 was determined (data not shown). A dose-dependent relationship was observed between the concentration of SEA used to stimulate the clone in the first stage of the assay and the reduced response to antigenic stimulation in the second stage (Figure 3). However, the A.E7 clone stimulated with SEA (up to 100 ng/mL) in the first stage maintained an essentially normal proliferative response to exogenous IL-2 stimulation in the second stage. These studies were carried out with a two day resting period between the stimulation with peptide antigen or SEA (stage 1) and the analysis of T cell function (stage 2). Additional experiments were carried out with up to seven days between the end of SEA stimulation and the initiation of the second stage, and we observed essentially the same degree of anergy (data not shown). Cells anergized under the latter conditions also retained normal responsiveness to IL-2. 

### 2.3. Expression of TCR-proximal signaling components in anergized A.E7 cells

**Figure 4:**
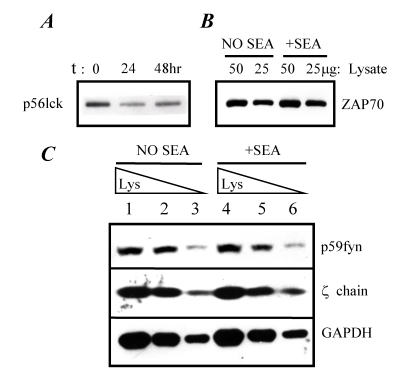
Reduced p56LCK expression in SEA-anergized A.E7 cells. A.E7 cells were stimulated with or without SEA (25 ng) for various times. Panel A, lysates were generated and analyzed (50 μg) for p56lck; Panel B, A.E7 cells were treated with SEA for 48 hours and lysates were analyzed for ZAP-70; Panel C, A.E7 cells were treated with SEA for 48 hours and lysates were analyzed for p59fyn, zeta chain, and GAPDH. Lanes 1 and 4 were loaded with approximately 150, 100, and 20 μg of lysate, respectively; Lanes 2 and 5 were loaded with approximately 100, 50, and 10 μg of lysate, respectively; Lanes 3 and 6 were loaded with approximately 50, 25, and 5 μg of lysate, respectively. These results are representative of three independent experiments.

Based on our previous results, superantigen-induced anergy in murine A.E7 T cells did not appear to be due to the simple failure of the stimulated T cells to produce IL-2 [19]. Furthermore, we previously showed that the anergy was not the result of TCR modulation [19]. However, we considered the possibility that the signaling components of the TCR may be altered as a part of inducing an anergic state. In an effort to address this question, the A.E7 clone was stimulated for 24 and 48 hours with or without SEA (25 ng/mL) in the absence of APCs. The A.E7 clone was harvested, washed, and lysed. The lysates were separated by SDS-PAGE and probed with antibodies specific for TCR-proximal signaling components. For comparative purposes within an assay, a volume of whole cell lysate from an equivalent number of cells was added to each lane. The results show that SEA-stimulation for either 24 or 48 hours resulted in a decrease in the expression of p56lck in the A.E7 clone (Figure 4A). Densitometric analysis of these bands revealed a 40% decrease in p56lck expression relative to the unstimulated control. These data are consistent with previously reported observations of anti-CD3-induced anergy [29]. Lck expression in the A.E7 clone was also analyzed after stimulation for 48 hours with SEA in the presence or absence of exogenous IL-2 (20 U/mL). Stimulation with the combination of SEA and IL-2 did not alter the 40% decrease in p56 lck expression that was observed with SEA stimulation alone (data not shown). Moreover, IL-2 stimulation alone did reduce the expression of p56lck by 50% (data not shown).

We also analyzed the expression of the TCR-proximal components p59fyn, ZAP-70, and p16 zeta chain (Figure 4B and Figure 4C). Again, the A.E7 clone was stimulated with and without SEA (25 ng/mL) for 48 hours, and the lysates were separated by SDS-PAGE and immunoblotted with antibodies specific for either ZAP-70, p59fyn, or zeta chain. The expression of these signaling components in the A.E7 clone was not altered by stimulation with SEA for 48 hours. The observation that p59fyn expression was not altered is in contrast to observations reported for anti-CD3-induced anergy where p59fyn expression dramatically increased [29]. As a control for these experiments, analysis of expression of the housekeeping protein, glyceraldehyde-3-phosphate dehydrogenase (GAPDH), was carried out as an additional means of normalization of lysates loaded in each lane. These results show that stimulation of the A.E7 cells for 48 hours with SEA did not affect the expression of GAPDH in the A.E7 cells (Figure 4C). The analysis of GAPDH for the purposes of normalization was performed in every assay. These results demonstrate that, with exception of p56lck, the components of the TCR-proximal signal transduction pathway were present at a normal level at 24 to 48 hours in the SEA-anergized A.E7 cells. 

### 2.4. TCR-proximal signaling components during the induction phase of anergy

**Figure 5:**
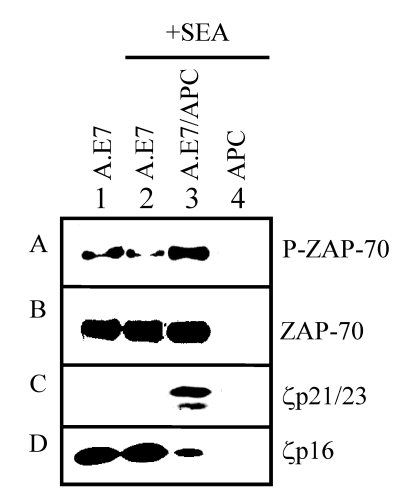
Uncoupling of TCR zeta chain during superantigen-induced anergy development. A.E7 cells were cultured with medium (lane 1), SEA (lane 2), or SEA presented by APCs (lane 3) for 30 minutes. As a control, APCs were also cultured with SEA (lane 4) for 30 minutes. APCs were depleted of T cells and pre-treated with SEA (25 ng/mL) for 30 minutes prior to addition of A.E7 cells. Lysates were prepared and analyzed by western blotting with anti-phosphotyrosine (Box A), anti-ZAP-70 antibody (Box B), or anti-zeta chain antibodies (Box C). Lysates in box A and C were immunoprecipitated with anti-CD3ε antibody followed by immunoblotting with anti-phosphotyrosine antibody. These results are representative of three independent experiments.

Published results have demonstrated that TCR-proximal tyrosine kinase signaling may be altered early during the induction of anergy [16,29,30,31,32]. For this reason, we measured the expression of TCR-proximal signaling components in the A.E7 clone stimulated with SEA (25 ng/mL) in the presence or absence of APCs for 30 minutes (Figure 5). We show in Figure 4 that ZAP-70 expression levels were not altered by SEA stimulation. In Figure 5 (Box B), we confirm this and demonstrate that ZAP-70 expression is also not changed in the A.E7 cells following stimulation under normal activation conditions (lane 3) when compared to anergic stimuli (lane 2). However, the phosphorylation state of ZAP-70 was increased with normal activation stimuli but not with anergizing stimuli (SEA alone) (Figure 5, Box A). These results suggest that either ZAP-70 is not recruited to the TCR or that ZAP-70 does not become phosphorylated by SEA in the absence of APCs. These data are consistent with observations reported for anergy induction using anti-CD3 antibodies [11].

During normal T cell activation, the zeta chain is typically hyper-phosphorylated. Cells anergized using live APCs presenting altered peptide ligand (APL) have shown that under these conditions the zeta chain does not become fully hyper-phosphorylated [16,32]. These results are in contrast to the hyper-phosphorylation of the zeta chain observed following anergy induction via antigen presented by chemically-inactivated APCs [32]. In light of these results, we wished to determine whether the zeta chain became hyper-phosphorylated upon stimulation with SEA in the presence or absence of APCs. Lysates of the A.E7 clone cultured in medium or stimulated for 30 minutes with SEA, APCs, or APCs plus SEA were prepared and analyzed by western blotting for the non-phosphorylated form (p16) of zeta chain (Figure 5). We observed that expression of the non-phosphorylated form of zeta chain (p16) in the SEA-stimulated A.E7 cells was unchanged when compared to non-stimulated A.E7 cells (Figure 5, Lanes 1 *vs.* 2). In contrast, when APCs were present with SEA, there was a significant reduction of the non-phosphorylated form (p16) of zeta chain (Figure 5, Box D). This loss of p16 zeta chain could be due either to degradation of the protein or by hyper-phosphorylation which would change the apparent size during electrophoresis. To determine if p16 was hyperphosphorylated, immunoprecipitation with anti-CD3ε antibody was used to co-precipitate the zeta chain followed by western blotting with anti-phosphotyrosine antibodies (Figure 5, Box C). Bands representing the hyper-phosphorylated forms of zeta chain (p21 and p23) were observed only from lysates of A.E7 cells that were stimulated with normal activation conditions (SEA presented by APCs; Figure 5, Lane 3). Immunoprecipates from lysates of A.E7 cells that were not stimulated or stimulated with SEA alone (anergizing condition) did not contain the p21/23 bands. Taken together, these results indicate that a normal T cell activation stimulus can induce hyper-phosphorylation of the zeta chain while an anergizing stimulus (SEA alone) cannot, even though both stimuli induce a strong proliferative response in the A.E7 cells. Stimulation of T cells with SEA in the absence of APCs appeared to result in the uncoupling of the zeta chain from the TCR/CD3 complex suggesting the possibility that these altered signaling events may have been involved in the development of anergy. 

Our approach has been to examine the induction of T cell anergy using SEA treatment of the A.E7 clone *in vitro*, and it should be pointed out that several other investigators have demonstrated that this superantigen induces a state of anergy with administration *in vivo* [33,34,35,36]. We acknowledge that using a single clone is a limitation with these studies since it may or may not reflect completely the *in vivo* state. However, the A.E7 clone used here is one of the first and most extensively characterized T cell clones to study the state of anergy *in vitro*. This offers an advantage to studying TCR signaling events because comparisons between different anergic stimuli can be drawn. Likewise, the use of a superantigen to stimulate a clone allows for all of the cells within a population to respond both homogenously and maximally. This is in contrast to using naïve T cells since a single superantigen will likely only stimulate up to 20% of the T cell repertoire [3]. Finally, the ability of a superantigen to induce anergy in the absence of MHC class II bearing cells allows the signaling events to be isolated from any interference of costimulatory signaling molecules.

The induction of anergy by superantigens has been demonstrated by our lab and others [19,37,38,39]. In the present study, we observed a failure of ZAP-70 and zeta-chain to become phosphorylated in response to SEA stimulation during the induction phase of anergy. Similar observations were found *in vivo* after anergy was induced [37]. In this case, mice were primed with SEB, and a week later the TCR Vβ-specific T cells were re-challenged with SEB presented by irradiated splenocytes as a sources of antigen presenting cells. The SEB-primed T cells were not able to phosphorylate ZAP-70 or zeta-chain upon re-challenge. On the other hand, studies where SEB is used to induce anergy *in vivo* show that it is the CD4 memory, but not naïve, T cells which fail to bring ZAP-70 into the TCR zeta-chain complex for phosphorylation and subsequent activation [38]. These data, in combination with our *in vitro* observations, suggest that the mechanism of anergy induction by superantigens may share similar characteristics *in vitro* and *in vivo*. Furthermore, these data suggest that the induction of anergy *in vivo* by superantigens may occur in the absence of antigen presenting cells.

Another issue which may be important for SEA-induced anergy in the A.E7 clone is that p56lck levels are reduced. Fujimaki *et al.* [39] demonstrated that the interaction of CD45 and p56lck was suppressed in TSST-1-anergized thymic CD4+ cells and that this played a role in sustaining an anergic state. Since p56lck levels are reduced in our system, it is possible that a similar mechanism involving CD45 and p56lck interactions proposed the latter investigators is taking place. Regardless, it seems that there may be common processes for the induction of anergy by superantigens.

The induction of peripheral tolerance or anergy is an important phenomenon which has evolved to protect the host against the development of autoimmune responses. Both bacteria and murine viruses have developed superantigens as one way to evade the immune system by inducing T cell anergy [3,40]. It has become apparent that a state of anergy develops in a portion of the T cells following HIV infection, and this anergic state can reduce the antiviral immune response [41,42]. It is important to understand the mechanisms responsible for the induction of anergy by these micro-organisms. Furthermore, understanding the biochemical basis of anergy induction may also lead to the development of strategies for the treatment of autoimmune diseases, such as, multiple sclerosis and type-1 diabetes [43,44]. 

## 3. Experimental Section

### 3.1. Reagents

C3H/HeN (H-2^k^, I-J^k^) and B10.A (H-2^k^) mice were obtained from the National Cancer Institute (Frederick, Maryland). Staphylococcal enterotoxin A (SEA) was obtained from Toxin Technology (Sarasota, FL). The synthetic peptide 93–103 corresponding to amino acids 93 to 103 (DLIAYLKQATK) of pigeon cytochrome C (PCC) and the recombinant human interleukin-2 (IL-2) were generous gifts of Dr. Dennis Taub (NCI, Fredrick, MD). The rabbit polyclonal antiserum specific for the tyrosine kinase p56lck and the ζ chain were generous gifts of Dr. Alexander Tysgankov (Temple University, Philadelphia, PA). The mouse monoclonal antibody specific for tyrosine kinase p59 fyn was obtained from Santa Cruz Biotechnology, Inc. The mouse monoclonal antibodies specific for tyrosine kinase ZAP-70 and phosphorylated tyrosine residues were purchased from Transduction Laboratories (Lexington, KY). The mouse monoclonal antibody specific for glyceraldehyde-3-phosphate dehydrogenase (GAPDH) was purchased from Biodesign International (Kennebunk, ME). The murine T helper type-1 T cell clone, A.E7, was derived from B10.A mice by Dr. Ronald Schwartz at National Institute of Health (Bethesda, Maryland; [45]). This clone recognizes an epitope of PCC composed of amino acids 84 to 104 presented in the context of I-E^k^. In addition, this clone expresses the T cell receptor (TCR) Vα 11 allele and Vβ 3 allele. The A.E7 T cell clone was maintained in a tissue culture medium containing RPMI-1640 medium, 10% fetal calf serum, 2 mM glutamine, 0.1 mM non-essential amino acids, 1 mM sodium pyruvate, 50 μM β-mercaptoethanol, and 0.01 mg/mL gentamicin. The A.E7 clone was stimulated every seven days with 0.5 μM synthetic peptide 93–103 or 25 μg/mL of PCC presented by irradiated (1500 rads) B10.A splenocytes. Two days after stimulation of the clone, recombinant human interleukin 2 (IL-2) was added to the culture at a final concentration of 20 units/mL. Hybridoma 145-2C11 was obtained from ATCC and produces monoclonal hamster antibody specific for the ε chain of murine CD3. 

### 3.2. T cell activation

The A.E7 T cell clone was cultured (10^5^ cells/well) in a 96 well plate with irradiated (1500–2000 rads) B10.A splenocytes (2.5 × 10^5^ cells/well) and either peptide 93–103 or superantigen (SEA or SEB) at various concentrations. The cultures were incubated for two days at 37 °C in a humidified incubator with 5% CO_2_ and 1 μCi of [^3^H]-thymidine was then added in each well. The cells were harvested using an Inotech 96 well plate harvester onto glass fiber filter paper. The filters were subjected to liquid scintillation spectrometry.

### 3.3. Anergy induction

The technique for the analysis of anergic T cells was a modification of the method of Jenkins and Schwartz [12]. In the first stage of anergy induction, the A.E7 T cell clone (10^5^ cells/well) was cultured in 96 well plates with SEA for two days and the plates were washed a total of three times. The plates were recultured in the presence of medium for two additional days. The A.E7 T cell clone was then assayed for proliferative capacity in the second stage of the experiment. The cells were again washed in the manner described above and incubated with either medium alone, irradiated APC, irradiated APC plus antigen (10 μM), or IL-2 (20 U/mL) for two additional days at 37 °C. Proliferation was measured as described above. 

### 3.4. TCR signaling analysis

After stimulation with SEA for two days in culture, the A.E7 cells were centrifuged at 250 g for 10 minutes at 4 °C. The pellets were resuspended in PBS and washed once. The pellets were lysed using TNE lysis buffer containing 10 mM Tris-HCl pH 7.6, 50 mM sodium chloride, 0.5% NP-40, and 5 mM EDTA, 10 μg/mL aprotinin, 1 mM sodium orthovanadate, 1 mM PMSF, and 5 mM sodium fluoride for 10 minutes on ice in a 1.5 mL polypropylene eppendorf tube. The lysates were centrifuged at 11,000 g for 4 minutes at 4 °C. The supernatants were collected and assayed for total protein concentration using the BCA Protein Assay kit. Between 100 μg and 2 mg of total protein for each immunoprecipitation was set aside. Either polyclonal antiserum for p56 lck or for ζ chain or purified antibody specific for p59 fyn and CD3ε was added to the whole lysate in a final volume of 500 μL of TNE lysis buffer and incubated at 4 °C for 1 hour. Fifty μL of a 10% pansorbin suspension was added to the mixture and incubated for 1 hour at 4 °C on an orbital rotator. The samples were then centrifuged for 1 minute at 11,000g at 4 °C and the supernatant decanted. The pellet was washed 3 additional times under these conditions with TNE buffer in the absence of protease inhibitors. The pellets were incubated 15 to 30 minutes with 40-50 μL of sample buffer containing 20 mM Tris-HCl pH 6.8, 4% sodium dodecyl sulfate, 20% glycerol, 10% β mercaptoethanol, and 0.006% bromophenol blue. The samples were centrifuged at 11,000 g for 1 minute, and the supernatants were removed, boiled 3-5 minutes, and loaded on a 10% SDS-polyacrylamide gel (SDS-PAGE). Western blot analysis was performed and the nitrocellulose paper was blotted with either antibodies specific for p56 lck, p59 fyn, ZAP-70, phosphotyrosine residues, or ζ chain.

The technique for the analysis of early TCR-proximal signaling events was a modification of the method of [46]. Briefly, splenocytes from B10.A mice were depleted of T cells as described above. These depleted splenocytes were cultured in A.E7 TCM in 24 well plates at a density of 1–1.5 × 10^7^ cells/well in the presence or absence of SEA at 100 ng/mL final concentration for 30 minutes at 37 °C. The A.E7 clone was then added at a density of 1–1.5 × 10^7^ cells/well in a final volume of 1.5 mL. The plates were centrifuged at 250 g for 3 minutes in order for the A.E7 cells to make contact with the APC monolayer, and were then incubated for either 10, 20, or 30 minutes at 37 °C. The cultures were harvested, the cells were centrifuged at 250 g for 10 minutes, and the supernatants were decanted and the pellets lysed for 10 minutes on ice with 200 to 300 μL of TNE lysis buffer containing the protease inhibitors described above. Twenty to 30% of lysate was reserved for immunoblotting analysis. The remaining lysate was used for immunoprecipitation purposes as described above. The content of the immunoprecipitates was analyzed by western blot analysis.

## 4. Conclusions

Superantigens exhibit both stimulatory and suppressive immunomodulatory properties. Here we report the proximal signaling events of the TCR in a murine T cell clone during the induction of anergy by SEA in the absence of MHC class II bearing cells. Under these conditions, SEA was able to induce unique signaling events when compared to experimental systems employing APCs. SEA stimulation alone failed to induce hyper-phosphorylation of the zeta chain despite inducing a strong proliferative response. These data suggest that superantigen induction of anergy involves an uncoupling of the TCR zeta chain function. Furthermore, these data suggest that superantigen ligation with the TCR offers a simplified model system of anergy induction where complicating signals provided by the APC are absent.
